# Hospitals, Schools, and Boys and Girls

**Published:** 1887-01-22

**Authors:** 


### HOSPITALS, SCHOOLS, AND BOYS AND GIRLS.

Many persons who would willingly befriend a hospital, but do not quite see how to do so, may find a useful hint in the following :—The lady principal of a private school in our town, having become interested in our Cottage Hospital, quietly arranged a sale of work in her school before breaking up for the Christmas holidays. The result was a nice little lump of £12 handed over to our treasurer. As the pupils were not very numerous, and the majority of them young, I think this says much for the industry and goodwill of all concerned. Not only schools, but many a private family where there is a large number of young people—boys could do their share as well as girls—and a good circle of acquaintances, might in this way give valuable help to struggling institutions. District Nurse.

